# Role of Ionic Strength in the Formation of Stable Supramolecular Nanoparticle–Protein Conjugates for Biosensing

**DOI:** 10.3390/ijms23042368

**Published:** 2022-02-21

**Authors:** Giorgia Brancolini, Vincent M. Rotello, Stefano Corni

**Affiliations:** 1 Institute of Nanoscience, CNR-NANO S3, via G. Campi 213/A, 41125 Modena, Italy; stefano.corni@unipd.it; 2 Department of Chemistry, University of Massachusetts, 710 North Pleasant Street, Amherst, MA 01003, USA; rotello@chem.umass.edu; 3 Department of Chemical Sciences, University of Padova, via Marzolo 1, 35131 Padova, Italy

**Keywords:** molecular dynamics, multiscale modeling, ionic strength, biosensors, functionalized metal nanoparticles, supercharged GFP

## Abstract

Monolayer-protected gold nanoparticles (AuNPs) exhibit distinct physical and chemical properties depending on the nature of the ligand chemistry. A commonly employed NP monolayer comprises hydrophobic molecules linked to a shell of PEG and terminated with functional end group, which can be charged or neutral. Different layers of the ligand shell can also interact in different manners with proteins, expanding the range of possible applications of these inorganic nanoparticles. AuNP-fluorescent Green Fluorescent Protein (GFP) conjugates are gaining increasing attention in sensing applications. Experimentally, their stability is observed to be maintained at low ionic strength conditions, but not at physiologically relevant conditions of higher ionic strength, limiting their applications in the field of biosensors. While a significant amount of fundamental work has been done to quantify electrostatic interactions of colloidal nanoparticle at the nanoscale, a theoretical description of the ion distribution around AuNPs still remains relatively unexplored. We perform extensive atomistic simulations of two oppositely charged monolayer-protected AuNPs interacting with fluorescent supercharged GFPs co-engineered to have complementary charges. These simulations were run at different ionic strengths to disclose the role of the ionic environment on AuNP–GFP binding. The results highlight the capability of both AuNPs to intercalate ions and water molecules within the gold–sulfur inner shell and the different tendency of ligands to bend inward allowing the protein to bind not only with the terminal ligands but also the hydrophobic alkyl chains. Different binding stability is observed in the two investigated cases as a function of the ligand chemistry.

## 1. Introduction

Monolayer-protected gold nanoparticles (AuNPs) have been extensively investigated to disclose their unique and diverse properties. [1,2,3,4,5] AuNPs have already shown great potential in applications, including chemosensing (i.e., small molecule detection in solution) [6,7], biosensing [8,9,10], catalysis (nanozymes) [11,12,13] and transport of chemical species in biological environments and cells (e.g., drug delivery) [14,15,16]. Inorganic nanoparticles can be engineered to possess physiochemical properties for specific applications, e.g., their shape [17] and size [18] can be tuned to define nanoparticle properties. The molecular recognition properties of these particles are dictated by the chemical structure of the coating ligands, which form self-organized and multivalent binding sites for the guest species [19], a feature crucial for nanoparticle colloidal stability [20,21]. The surface of the nanoparticles interfaces with the external environment, and appropriately engineered surfaces can be used to regulate interactions between nanoparticles and biomolecules [22,23,24] driven by non-covalent interactions [9,25].

The role of surface curvature and ligand composition of AuNPs of various sizes has been systematically investigated by atomistic simulations [26,27,28,29]. Metallic particles with core size below 3 nm exhibit molecule-like properties. This feature expands their potential use to the development of sensors and platforms for therapeutics delivery traditionally reserved for small-molecule medicine. A deeper insight into AuNP–bio interactions can be gained with a judicious combination of in vitro experiments and computer simulations. However, so far, the ability to control the behavior of those ultrasmall NPs is limited, partly because of limitations in chemical synthesis and partly because of our incomplete understanding of their interactions in biological environments [30,31,32]. The present study addresses the second limitation. 

Functionalized nanoparticles are able to act as flexible scaffold for interfacing with proteins [33,34,35], but environmental factors, such as solvent, temperature, ionic strength, intercalation of ions/surfactants on the surface of NPs, can act as a potential challenge [36], especially in the field of NP-based biosensors. On the other hand, the local environment around NPs can, in principle, be employed also to control the interactions of NPs with proteins. 

Supramolecular recognition between proteins and nanoparticles has been extensively studied through MD simulations [37,38,39,40]. The prediction of the electrostatics of supramolecular protein–NP aggregates is far from trivial, since many environmental factors can contribute to the stability of the final assemblies. The local environment of NPs has a profound interaction on the surface properties of NPs, which alter the physico-chemical properties of both the environment and the surface. The surface of NPs creates a special local environment, with different properties compared with the bulk [41]. This unique environment has strong effects on NP-based sensors, as those sensors probe concentrations of the local environment, and not from the bulk. In the other direction, the surface charge of NPs is determined by the presence of ions in the local nano environment. Through a reaction with ligands or even with the bare NP surface, this environment regulates the surface charge of NPs and thus also their colloidal stability. 

In this article, we focus on a recently developed method for co-engineering gold nanoparticles and proteins, which has been shown to provide enhanced binding affinity under conditions of physiologically relevant ionic strength, which normally disrupts particle–protein interactions [42]. We perform atomistic simulations to disclose the role of the local distribution of ions within the AuNPs monolayer in specific and experimentally well-characterized Au_144_NPs (2 nm core diameter), namely functionalized with cationic arginine-terminal ligand (AuArg) and with a negatively charged amino acid (Asp/Glu), i.e., AuCOO, respectively. Cationic arginine-based ligands are used to generate a multivalent nanoparticle host to form a robust complex with highly negatively charged GFP (−30GFP). −30GFP is a variant of GFP in which 15 surface-exposed residues are mutated to negatively charged amino acids (Asp/Glu) [42]. Similarly, highly positively charged +36GFP is co-engineered to interact with anionic AuCOO [43]. Here, we use atomistic simulations to disclose the role of the ionic environment around experimentally synthesized charged monolayer-protected gold NPs in determining their physico-chemical properties and their interactions with “supercharged” proteins of complementary charges, designed to act as “chemical nose” biosensors [44].

By means of extensive atomistic simulations, we disclose the local ionic nano environment around monolayer-protected AuNPs, which modulates their binding with supercharged GFPs [45,46]. Multiple molecular dynamics simulations were performed at NaCl solution concentrations of 50 mM, 100 mM and 200 mM. Simulations were performed at neutral pH (pH = 7.4), according to the experiments (i.e., the protonation state of titrable residues was chosen coherently to neutral pH). For each ionic concentration, four different initial random distributions of ions were sampled in evaluating the interaction.

From our simulations using state-of-the-art force fields for the AuNPs, we observe the ability of ions and water molecules to intercalate within the AuNPs monolayer, indicating that, for AuNP-based sensing applications, the actual ion concentrations at the NP surface are different from bulk values, and the affinity of the AuNP for the complementary protein increases with increasing ionic strength, as observed in experiments [47]. Namely, the experimental formation of discrete nanoparticle–protein assembly was previously observed with dynamic light scattering (DLS), and the corresponding binding affinities of the AuNPs host with the GFP guests were quantified with fluorescence titrations by parametrically varying salt concentration (NaCl, 0–200 mM) in 5 mM phosphate buffer. Simultaneously, rigid body docking studies were performed at increasing ionic strengths to shed light on the fundamental forces involved in binding, but the ligand conformational dynamics and the ion and water molecules intercalation dynamics were ignored.

Here, by means of fully flexible MD, we observe that the intercalation of charges within the AuNPs monolayer is due to different conformation of the ligands, which modifies the overall charge experienced by the GFP during the binding. As a consequence, the stability of protein–AuNP conjugates can be maintained at comparable stability even at different ionic strengths due to the number of ions trapped within the monolayer at different ionic strengths, which differs from screening due to the solution. The ion distribution around the NP mixed monolayer is also observed to participate in the binding with protein.

The present atomistic modeling aimed at achieving an efficient sampling and exploration of the configurational space of molecular motifs at different ionic concentrations provides a promising platform for the creation of bioconjugate protein–NP supramolecular complexes that are stable at physiologically relevant conditions of higher ionic strength, enhancing their practicality for applications in biological contexts [48].

## 2. Results and Discussion

### 2.1. Investigated Systems

**Figure 1:**
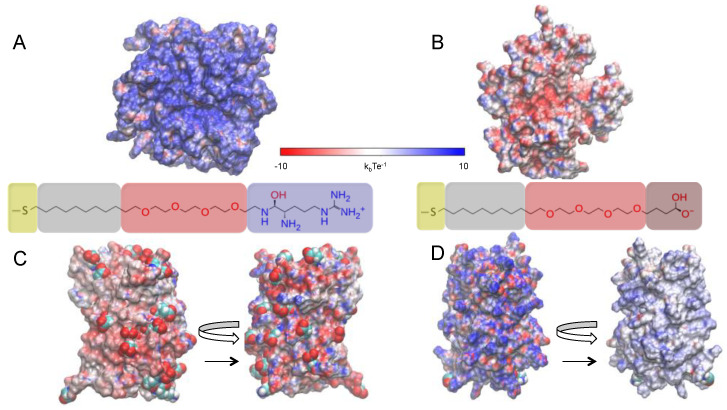
The monolayer-protected gold nanoparticles and proteins investigated in this study. Panel (**A**) Electrostatic potential surface of the AuArg (blue: positive charge; red: negative) and scheme of the AuNP functionalized ligands. Panel (**B**) Electrostatic potential surface of the AuCOO and scheme of the AuNP functionalized ligands. Panel (**C**) Electrostatic potential surface of −30GFP engineered protein with complementary charge in respect to AuArg. Panel (**D**) Electrostatic potential surface of +36GFP engineered protein with complementary charge in respect to AuCOO.

In this work, we use atomistic simulations to disclose how the binding interactions between two supercharged GFPs and two engineered AuNPs (see Figure 1) are affected by the ions present in the environment. Two types of ligands are considered for the monolayer-protected AuNPs, namely AuArg (i.e., Au_144_ [L_60_]^+60^ [L = S(CH_2_)_9_(OC_2_H_4_)_4_Arg^+^]) (Figure 1A) and AuCOO (i.e., Au_144_ [L_60_]^−60^ [L = S(CH_2_)_9_(OC_2_H_4_)_4_COO^−^]) (Figure 1B). The complementary protein to interact with AuArg is a highly negatively charged GFP (−30GFP) in which 15 surface-exposed residues are mutated to negatively charged amino acids (Asp/Glu) (Figure 1C). Instead, +36GFP is the model protein to interact with AuCOO, which is obtained by mutating 29 residues of *wt*GFP into positively charged amino acid residues (Lys/Arg) (Figure 1D). More details on GFP sequences can be found in the Supplementary Material.

### 2.2. Effects of Ions on Single AuNP Nanoparticles

The initial structures for the AuNP MD simulations were obtained from nanomodeler [49], and the ligand conformations around the nanoparticle were studied in the presence of water environment and counterions at ionic strengths of 50 mM, 100 mM and 200 mM NaCl.

The data were analyzed in terms of the radial distribution functions (RDFs) calculated for the terminal ligand atoms, but also for the surrounding Na+ and Cl− ions and for the water molecules, respectively. In Figure 1A, the blue lines show the RDF computed for the center of mass (COM) of the AuArg guanidinium group (defined as the three nitrogen terminal atoms) with respect to the COM of the gold core; the red lines indicate the distribution of the main Cl− ions, the green lines that of Na+ ions and the black lines that of the water molecules. In Figure 1B, the brown lines refer to the RDF computed for the COM of the AuCOO carboxylic groups (defined as the two terminal oxygen atoms); the green lines refer to the main Na+ ions, the red lines to the Cl− ions and the black lines to the water molecules, respectively.

**Figure 2:**
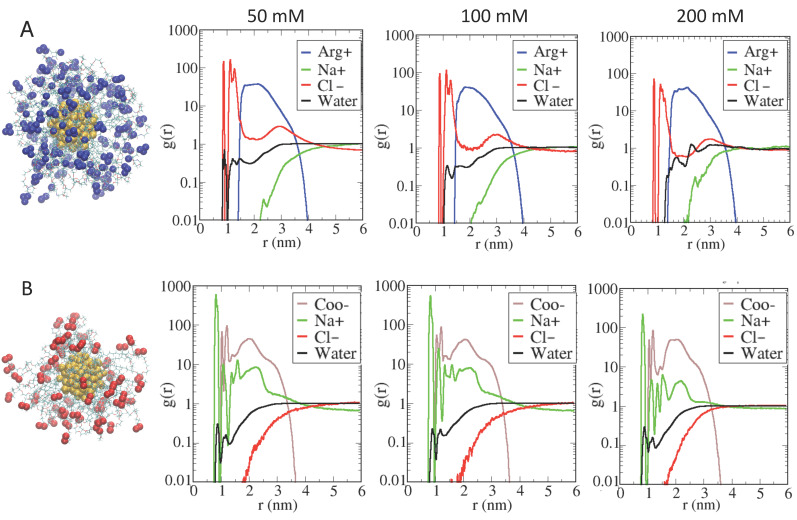
Simulation snapshots of both AuNPs with Au_144_ gold core. Panel (**A**) shows a positively charged 60+ particle with cationic arginine-terminal ligand (depicted with blue balls), namely AuArg, whereas Panel (**B**) shows a negatively charged −60 particle with anionic glutamate-terminal ligand (depicted with red balls), namely AuCOO. The plots identify the radial distribution functions at three different ionic strengths for AuArg and AuCOO, respectively. Distances are measured relative to the center of mass of the gold core with respect to the center of mass of the terminal ligand atoms and to the different ions or water molecules. For each AuNPs, the plots indicate similar features at increasing ionic strength, highlighting the presence of counterion density at contacting distances with the gold within the monolayer.

Figure 2A,B provide a detailed insight into the structuring of the monolayer interface for the two types of AuNPs at different ionic strengths.

The RDF behavior is similar for both AuNPs. As to the behavior due to AuNP, the radius of the inner gold core is located at 0.65 nm, whereas the radius of the gold–sulfur outer shell is 0.9 nm (see SI). In Figure 1A (for AuArg), the contribution of the main Cl− ions (red line) shows a peak decaying to 0 at a distance of about 0.9 nm, with a nearby first maximum corresponding to a density of Cl− ions directly interacting with the gold–sulfur shell, while a second peak is centered at distances farther from the gold surface, namely from 1 to 1.5 nm, which corresponds to a density of Cl− ions interacting directly with the alkane–PEG ligands. A third broad peak of Cl− ions is found at distances of about 2.9 nm, near the Arg end group, which is consistent with an increased Cl− counterion density in the region outside the AuNPs, as expected. Conversely, an increase in Na+ density (green line) at a larger distance is observed in the outer region. It is interesting to note that both Cl− ions and water molecules are able to penetrate within the AuNP monolayer toward the gold surface, modifying the total net charge of the AuArg. This observed behavior can be interpreted on the basis of our refined AuNP model in which a charge distribution on the surface of gold atoms is explicitly evaluated, and the flexibility of the grafted sulfur head groups of the thiol ligands is allowed during the simulations. In addition to water and Cl− ions, a unique broad peak for the Arg end group (blue line) is centered at around 2.1 nm, which reflects the flexibility of the tails as observed in simulation snapshots. The broad peak for the ligands at 50 mM indicates a larger flexibility of the terminal groups at lower ionic strength relative to that at the higher ionic strength. Namely, an increase in ionic strength has the effect of partially reducing the overall flexibility of the ligands associated with a higher charge repulsion with an increased density of Na+ ions. As a consequence, the overall radius of the AuArg monolayer is reduced.

The enhanced peak for Cl− ions near the Arg end groups is consistent with a counterion condensation in this region. The qualitative features of RDF plots computed at increased ionic strength are largely identical and differ mainly at 200 mM, reflecting an increased density of Na+ ions (centered at around 2.5 nm) and a slightly decreased density of water molecules within the monolayer.

In Figure 2B, the AuCOO snapshot is reported together with the RDFs. The RDF profile of the carboxylate end group reveals substantial differences with respect to those obtained for AuArg. The RDF profile of the carboxylic negatively charged end groups (brown line) resides relative to the interface at distances of about 1 to 1.4 nm, indicating the clear tendency of a fraction of ligands to fold toward the gold surface to interact directly with some gold surface atoms (at around 1 nm) together with the Na+ ions and water molecules that have penetrated within the monolayer. A second relative maximum at around 2 nm indicates a fraction of ligands only partially folded toward the gold core and fluctuating around a distance near 2 nm, which corresponds approximately to the average radius of AuCOO. A third maximum at around 2.9 nm indicates a density of ligands in a stretched (or elongated) conformation. Two sharp peaks of Na+ ions (green line), at around 0.8 nm and 1.1 nm, are observed. The inner density reflects a fraction of Na+ ions interacting directly with gold core atoms. We note that in the case of AuCOO, a density of the main counterion Na (r = 0.098 nm) is observed at distances that are not accessible to the Cl− ions due their larger ionic radius (r = 0.181 nm). A second Na+ ion density is observed at around 1.1 nm, indicating Na+ ions interacting with sulfur head groups of the thiol ligands. Instead, a broad distribution of Na+ of around 1.3 nm to 3.6 nm indicates counterions condensed in the vicinity of charged end groups, lying in differently elongated conformations that correspond to different lengths, thus generating an overall anisotropic distribution of ligands around the AuCOO, which is not observed in the case of AuArg. A Cl− ion density (red line) is observed at farther distances, as expected. Qualitatively, at increasing ionic strength, no major changes are observed, except for a slightly broader density of Na+ counterion around the gold surface at 100 mM, which is instead at 200 mM and is largely identical to that observed at 50 mM.

To summarize, in the case of AuArg, the ligands tend to bend inward, reducing the ligand lengths associated with the peaks as a function of the increasing ionic strength. These results are in agreement with experimental DLS data previously obtained for the same particles at increasing salt concentrations (from 0 mM to 100 mM), providing a slight reduction in hydrodynamic radius for both AuArg and AuCOO in solutions of high ionic strength [42].

This bending is stronger in AuArg with respect to AuCOO, since the latter preserves a larger final radius and still quite a spherical shape, while anisotropies are evident in the case AuCOO, displaying mass distribution in three distinct lobes, which is more pronounced at low ionic strength but still observed at higher ionic concentration.

### 2.3. Role of Ionic Strength on Supramolecular Assemblies of Nanoparticles and Proteins

Initial supramolecular assemblies between AuNPs and GFPs protein are taken from the outcomes of a series of Browian Dynamics (BD) simulations performed at 50 mM, 100 mM and 200 mM NaCl solution concentrations, respectively [42]. 

**Figure 3:**
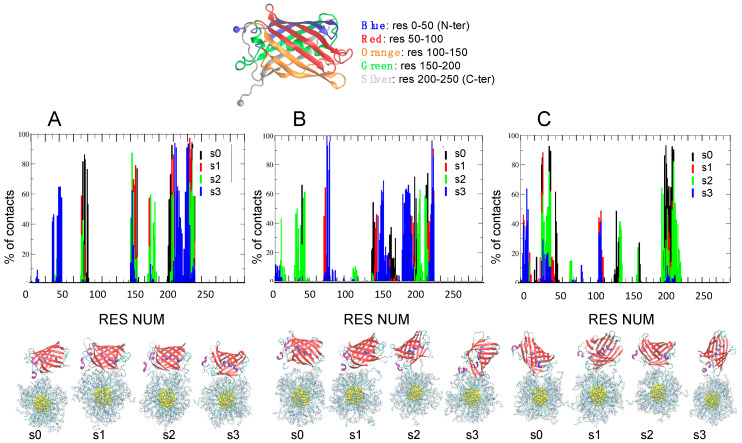
On top, the protein color scheme is used to label amino acid numbers along the protein secondary structure. In Panels (**A**–**C**), the plots report the contacting residue numbers versus the percentage of contacts in frames at the ionic strength of 50 mM, 100 mM and 200 mM. For each ionic strength, the simulations are repeated four times with different initial seeds of s0, s1, s2, s3, respectively. On bottom, −30GFP-AuArg binding orientations are represented at all seeds (proteins are depicted in ribbon representation and the AuArg in ball and sticks representation).

**Figure 4:**
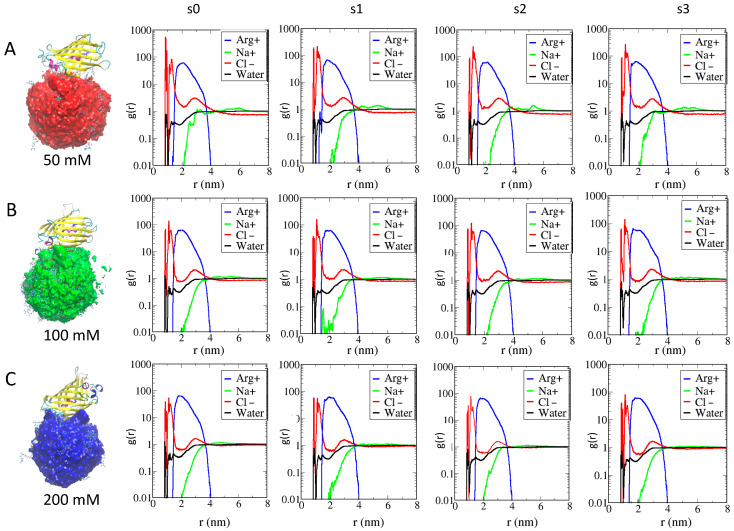
Panels (**A**–**C**) report the volume map for the AuArg when it is bound to the −30GFP (on the left) and the RDFs at three different ionic strengths (on the right), respectively. RDFs are measured relative to the center of mass of the gold core with respect to the center of mass of the terminal ligand atoms of Arg (blue line), to the different ions (Na+ in green and Cl− in red) and water molecules (black line). For each AuNPs, the plots indicate similar features at increasing ionic strengths, highlighting the presence of counterion density at contacting distances with the gold within the monolayer.

**Figure 5:**
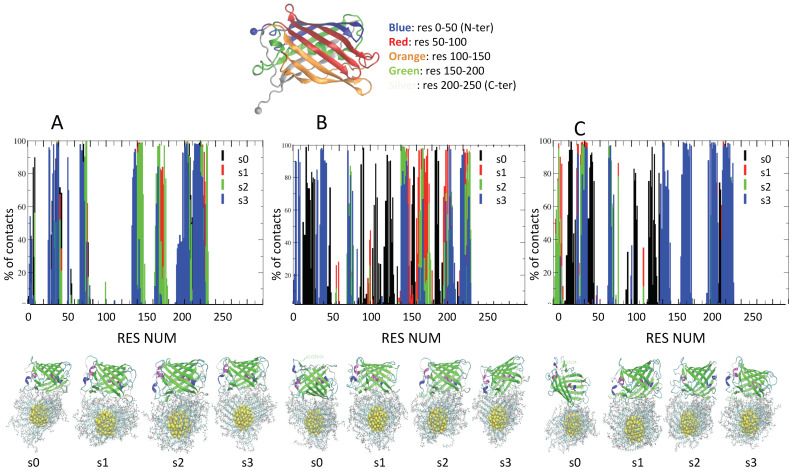
On top, the protein color scheme is used to label the amino acid numbers along the protein secondary structure. In Panels (**A**–**C**), the plots report the contacting residues numbers versus the percentage of contacts in frames at the ionic strengths of 50 mM, 100 mM and 200 mM. For each ionic strength, the simulations are repeated four times with different initial seeds of s0, s1, s2, s3, respectively. On bottom, +36GFP-AuCOO binding orientations are represented at all seeds (proteins are depicted in ribbon representation and the AuCOO in ball and sticks representation).

**Figure 6:**
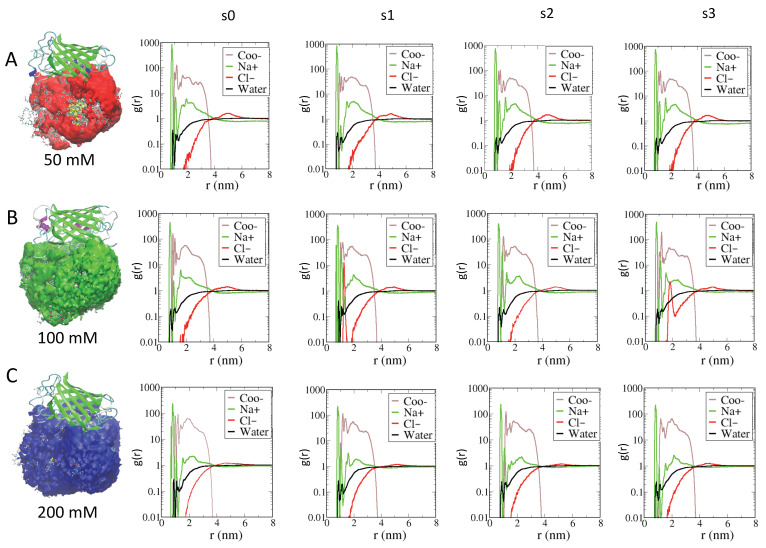
Panels (**A**–**C**) report the volume map of AuCOO when it is bound to +36GFP (on the left) and the RDFs at three different ionic strengths (on the right), respectively. RDFs are measured relative to the center of mass of the gold core with respect to the center of mass of the terminal ligand atoms of COO (brown line), to the different ions (Na+ in green and Cl− in red) and water molecules (black line). For each AuNPs, the plots indicate similar features at increasing ionic strengths, highlighting the presence of counterion density at contacting distances with the gold within the monolayer.

To improve the sampling over the distributions of Na+ and Cl− ions in solution and at the interface between GFP and AuNP upon binding, for each complex, MD simulations were repeated four times at each ionic strength, using different initial distribution of ions within the simulation box. Four different seeds are used (s0, s1, s2, s3) to start a total of 12 independent simulations of 200 ns. The results are summarized in Figure 3 and Figure 4 for −30GFP/AuArg and in Figure 5 and Figure 6 for +36GFP/AuCOO. The intermolecular energy decompositions among all the possible conformations sampled by AuArg were computed over the trajectory frames and are reported in the Supporting Information (Figures S1 and S2). Then, the intermolecular van der Waals and Coulomb interactions were estimated using the rerun Gromacs option on the frames. The outcome of the MD simulations is discussed below, referring to Figure 3 and Figure 4 and Table 1 for −30GFP/AuArg complexes and Figure 5 and Figure 6 and Table 2 for +36GFP/AuCOO, respectively.

***−30GFP/AuArg complexes.*** The resulting orientations of the supramolecular assemblies after 200 ns MD at T = 300 K, and the percentage of contacts found in all frames, are reported in Figure 3. Histograms indicate the percentage of frames in which a given residue is in contact (distance < 4 A) with AuArg atoms. From the plot, we can see that once a reasonable number of contacts are formed, they tend to be maintained during the dynamics run. However, with increasing ionic strength, some protein-contacting residues becomes more weakly bound to the surface of the AuArg, even if a global detachment of the protein from the monolayer is never observed. We remark that once the supramolecular complexes are formed, there is no unbinding event occurring at the interface between the AuArg monolayer and the −30GFP residues, even if at higher ionic strength, the formation of more transient binding patches is observed more frequently with respect to the formation of stable networks across the monolayer.

The binding patches obtained for the protein in multiple runs are differentiated by color in Figure 3, based on the different initial seeds (black for s0, red for s1, green for s2 and blue for s3). To investigate the percentage of long lifetime contacts established in each case, in Table 1 we report the −30GFP binding patches, which have the highest percentage of contacts (>70%) in all frames.

**50 mM:** in two cases out of four, namely s0 and s1, a large number of hydrophobic anchoring groups (TYR149, TYR198, PHE221) and negatively charged residues (ASP196, ASP228, ASP147, GLU202) form stable contacts. In s0, the strong fluctuations and the strong and specific binding occurring between the C-terminal tail within the monolayer during MD allow −30GFP to reorient to a new orientation, with the tail penetrated within the monolayer and an α-helix close to the surface (resnum 74–79). The binding of −30GFP in this orientation is stabilized by a single contact via ASP228 and strengthened by the C-terminal residues (PHE221, THR223, GLY226 ILE227) and the α-helix residues (PRO73, MET76, HIS75). In s1, the binding involves lateral β sheets of the protein, instead of the apical parts. The binding involves the residues of the C-terminal tail, but the orientation of the −30GFP is maintained stably during the simulation, and the interactions involve residues of the protein barrel. Both hydrophobic anchoring groups (TYR198, PHE221) and negatively charged residues (ASP147, GLU202, ASP228) stabilize the binding. In s2, the protein orientation is very similar to s1, but the monolayer ligand can attach and detach slightly more freely from the surface of the protein, which does not change its orientation during the entire dynamics. In s3, the binding occurs initially via the C-terminus, but strong fluctuations turn the tail away from AuArg surface and allow the protein to bind through a flexible loop that is stabilized by the residues GLU29, GLU31, GLU36, GLU38, THR40, LEU41. During the entire simulation, the C-terminal tail and the neighboring region is contacting the surface of the monolayer.

**100 mM:** in all cases, the −30GFP binding occurs with the apical part of the protein and not via the protein β barrel, which is more favorable to binding, since it is engineered to bear negatively charged residues. In s0, the initial orientation of the protein involves lateral β sheets, but the strong fluctuations of the C-terminal tail induce a global rotation of the protein with respect to the AuArg monolayer. The final complex binds the surface mainly through residues of the C-terminus, but it is strengthened by the binding with GLU222 and PHE221. In s1, the interaction occurs via the C-terminal region, which anchors the protein to the surface of the AuArg with terminal residues ILE227 HIS229.

In s2, the binding is stable but the residues interacting at the interface change during the simulation, and none is found in more than 70% of the frames. The protein initially interacts with the N-terminal and C-terminal region; it “walks” on the surface of the AuArg monolayer. At this ionic strength, it is not easy for the protein to re-orient in order to maximize its interaction through the residues of the barrel. Thus, no long-lived stable contacts are formed, and at the end of the simulation, the C-terminal tail is turned away, and instead the N-terminal region is binding at the short distances. In s3, the protein is anchored to the AuArg through the C-terminal residues, namely ILE227, HIS229, but during the simulation, the protein changes its orientation. This re-orientation also allows the N-terminal tail to bind the monolayer; however, the fluctuations turn away the N-terminus to the AuArg. Initially the protein interacts with its apical tails in a vertical orientation with respect to the surface of the monolayer, but after “walking” on the surface, it allows the C-terminal tail to enter the pocket on the monolayer, and the final binding occurs via the C-terminal (ILE227, HIS229) and the α-helix residues (HIS75, GLN78, HIS79), similarly to the complex s0 at 50 mM.

**200 mM:** In s0, the binding occurs initially via the C-terminus, but strong fluctuations turn the tail away from AuArg surface. These fluctuations allow the protein to bind through a flexible loop that is stabilized by the loop residues SER206, ASP208, PRO209, ASN210 and by residues belonging to the barrel namely GLU30, GLY31, THR41, LEU42, LYS43. During the entire simulation, the C-terminal and N-terminal tails are on the opposite side with respect to the surface of the monolayer. In s1, the interaction occurs via the barrel, mediated by GLU32 and THR41, but the protein is able to form long-lived contacts. The protein is able to spin and to change its interaction with the AuArg by keeping the apical regions far from the interface. N-terminal and C-terminal tails are not involved in the binding. The final complex S2 is very similar to complex s1. In s3, there is a global instability of the binding patch due to a continuous change of the binding interface inducing a global rotation of the protein with respect to the AuArg monolayer during the simulation. The final complex resulting from this simulation is binding the surface through the residues of the N-terminus.

To summarize, at low ionic strength, the protein is able to form binding patches, which in most cases involve the −30GFP engineered barrel, since binding involves the lateral beta sheets of the protein, which are negatively charged (see Figure 1C) By increasing ionic strength, the binding occurs mainly with the apical parts of the protein, namely mostly the C-terminal region, and in few cases, the N-terminal region. At the high ionic strength of 200 mM, the screening of ions allows the protein to undergo global rearrangements, which drives it to bind through different patches during the simulation. The results agree with nanomolar NP–protein binding affinity experimentally observed even at physiological ionic strengths [42].

RDF analysis on the AuNPs monolayer upon the formation of supramolecular assemblies at different ionic strengths is reported in Figure 3. When comparing the RDF of AuArg in the presence of the −30GFP protein with RDF without the protein (Figure 2A), we observed that in binding with the protein, a larger number of counterions and water molecules remain entrapped within the monolayer even at 100 mM and 200 mM, namely at the highest ionic strengths. Those counterions are lost when the AuArg is alone in the solution; they are participating in the binding of AuArg with the protein. Thus, their presence must be taken into account when designing AuNP of specific charge, since the overall “effective” charge of the AuArg is modified by the presence of the ions. Thus, the effects of the environment on the structure of the AuNP monolayer are related to the presence of a stable layer of counterions affecting the overall flexibility of the ligands and, hence, the electrostatics of interactions.

From Figure S1 in the Supporting Information, the intermolecular energy decomposition shows that even at high ionic strengths, supramolecular complexes, such as s3 at 100 mM and s0 at 200 mM, still have comparable binding energies with respect to complexes s0 and s1 at 50 mM formed at low ionic strengths.

***+36GFP/AuCOO complexes.*** A significantly different behavior is observed in the case of the AuCOO interacting with +36GFP. In all cases, the interaction is fast and the protein/AuNP binding is stronger. The protein always interacts with protein beta barrel residues, which are co-engineered to be of complementary charge with respect to the AuCOO monolayer. From the interaction of energy decomposition, it is possible to observe, for example, that complexes s3 at 200 mM and complexes s3 at 100 mM have comparable energetics with respect to s3 at 50 mM. The results indicate that for these dyads, the effect of the environment is very small, and the same binding interface can be conserved even in the presence of a high screening due to ions in the solution.

**50 mM.** In four cases out of four, the protein forms fast and stable contact with lateral β sheets on the side, which involves the C-terminal tail. The contact patches are very similar in s0, s1, s2 and s3, involving a large number of positively charged residues (LYS9, ARG37, LYS39, ARG71, ARG140, ARG2020, LYS204) and hydrophobic anchoring groups (TYR141, PHE143, PHE221).

**100 mM.** In three cases out of four, the binding occurs through the barrel side, which involves the C-terminal tail, and the binding patches are maintained the same as those of the protein at lower ionic strength of 50 mM. Thus, binding in s1, s2, s3 involves very similar contacting residues, and terminal residues of the C-terminus. In s0, the binding instead occurs through an opposite side of the barrel, which involves the N-terminal tail. The residues are different, and the binding is weaker, as supported by data reported in Table S2.

**200 mM.** In three cases out of four, the system interacts with lateral β sheets and in one case, the binding occurs through the apical part of the protein opposite to N-terminus and C-terminus. In s0, the binding occurs through the apical part with many positively charged residues involved in the binding (LYS124 LYS129 LYS122 LYS105 LYS24 LYS103 ARG126 LYS50 LYS212), and only one tyrosine TYR104 is involved. In s1 and s2, the binding involves the side of the beta barrel that involves the N-terminal tail, i.e., among others, ARG7, PHE6, LYS9. In s3, the binding occurs through the same barrel side that involves the C-terminal tail, and the binding patches are maintained the same as those identified for the protein at lower ionic strengths of 50 mM and 100 mM.

The effect of ionic strength on the RDF of AuCOO is similar to that observed for AuArg. The main effect on protein binding is to allow the presence of a stable and structured layer of ions and water molecules to remain trapped within the monolayer affecting the overall charge of the AuCOO interacting with +36GFP. The main difference in AuCOO versus AuAfg is due to the anisotropic distribution of ligands, which allow the presence of several pockets in the ligand shell to be created. Thus, the protein can interact more strongly with the AuCOO due to the possibility of forming more contacts at short distances.

To summarize, at low ionic strengths, the protein is able to form fast and stable binding patches involving the side of +36GFP engineered barrel, involving the lateral beta sheets and the C-terminus. By increasing the ionic strength, this identified binding is found to be robust even at 100 mM. At a high ionic strength of 200 mM, the screening of ions allows the protein to undergo global rotations, which drives it to bind through different patches during the simulation involving the lateral beta sheets and the N-terminus. Remarkably, the binding is maintained stable and still involves a large number of charged residues even at the highest ionic strengths, according to experimental data [42].

## 3. Materials and Methods

MD simulations of the fully hydrated single AuNPs in solution with water and ions were performed. For the AuArg and AuCOO, different ionic strengths were considered by changing the Na+ and/or Cl− counterion concentrations in various cases.

### 3.1. Development of Force Field Parameters for AuArg and AuCOO

OPLS/AA FF parameters have been developed for the AuNPs compatible with the previously developed GolP FF parameters for gold nanoparticles [12,47]. For the entire AuNP, the topology was generated using TPPmktop server [50], and OPLS/AA standard force field was used to parametrize the organic ligand monolayer atoms of the AuNP system, while the GolP LJ parameters, originally developed in the OPLS format, were used for Au and S atoms. SPC/E model was used to describe water molecules.

RESP (Restrained electrostatic potential) charges [51] were explicitly derived from all the atoms belonging to the smallest AuNPs surface repeating unit, namely AUS-SR-AUL-SR-AUS. Where SR was the alkanethiol functional group connected to the core through a sulfur atom forming the “staples” (see Figures S3 and S4 in SI), S was the sulfur atom forming a covalent bond with one gold atom at the interface (AUL = gold ligand) and a gold atom at the surface (AUS = gold surface) The charges were derived using R.E.D. server [52] at DFT level.

### 3.2. Simulations Methodology and Setup

MDs were performed with the GROMACS2020 software [50] MD simulations for both the single AuNPs and the conjugated complexes −30GFP/AuArg and +36GFP/AuCOO were subjected to an equilibration protocol, starting from 100,000 steps of steepest descent energy minimization, followed by 20 ns of NPT dynamics in which the solute and the solvent/ions were equilibrated separately. The Particle Mesh Ewald method [53] was used to treat the long-range electrostatic interactions, with the cutoff distance set at 10 Å. Short-range repulsive and attractive dispersion interactions were described by a Lennard-Jones potential, with a cut off at 10 Å. A time step of 2.0 fs was used, together with the LINCS [54] algorithm to constrain H-bonds. During the production phase, only gold core atoms of the NP were restrained, and the Bussi–Donadio–Parrinello [55] thermostat was used to maintain temperature at 300 K, and for pressure coupling at 1 bar, the Parrinello–Rahman [56] barostat was used. MD productions of 200 ns for the single NP and of 500 ns were performed for all the 12 complexes. The trajectories were analyzed in terms of density, temperature, potential energy and other macroscopic properties with Gromacs tools and with in-house scripts.

### 3.3. Setup of the Atomistic Simulations

The protein and AuNPs complexes obtained from previous BD simulations [42] were used as initial coordinate files for the MD simulations. Namely, rigid-body dockings were performed with SDA 7.2 [57] software to identify the initial possible adsorption orientations of −30GFP/+36GFP on the AuArg/AuCOO surface, respectively. Reduced charges were included in the docking to account for the number of Cl− counterions trapped within AuArg/AuCOO monolayer from MD simulation at each ionic strength (more details can be found in ref. [42]). The docking trajectories of protein–AuNP complexes were clustered, and the first most stable protein orientations were used as starting points for the MD simulations at each ionic strength.

In the case of a system with a single AuNPs, a cubic box with dimensions of (180 Å × 120 Å × 120 Å), including SPC/E water molecules and ions, was built. For AuArg at 50 mM, 79 Na and 139 Cl, at 100 mM 159 Na and 218 Cl, at 200 mM 317 Na and 377 Cl. For AuCOO at 50 mM, 138 Na and 78 Cl, at 100 mM 216 Na and 156 Cl, at 200 mM 372 Na and 312 Cl.

For the supramolecular complexes, including AuNP, protein, water and ions, a cubic box sized (180 Å × 180 Å × 180 Å) was built. The protein was placed at the center of the simulation box, and the AuNPs were kept as in the representative complexes of the docked clusters obtained from previous BD docking simulations. Before the addition of water molecules, the center of mass of the protein was translated by 5 Å from the center of mass of the AuNP to allow the addition of interfacial water molecules, but retaining the original docked orientation with respect to the AuNP. For the −30GPF/AuArg complexes at ionic concentration of 50 mM, 154 Cl and 123 Na were added, at 100 mM 278 Cl and 247 Na, at 200 mM 524 Cl and 493 Na, respectively. For the +36GFP/AuCOO complexes at ionic concentration of 50 mM, 148 Na and 123 Cl, 100 mM 272 Na and 247 Cl, 200 mM 518 Na and 493 Cl.

At each ionic concentration, four independent 500 ns runs, starting from the same supramolecular complex, were performed, each starting with different initial seeds (s0, s1, s2, s3) for the spatial distribution of ions in the simulation box.

## 4. Conclusions

Multiple parallel atomistic simulations of two oppositely charged monolayer-protected AuNPs interacting with fluorescent supercharged GFP co-engineered with complementary charges were performed. Simulations were performed and compared at different ionic strengths to disclose the role of the ionic environment on the binding.

Our first concern was to investigate the effect of increasing the concentration of the surrounding ions on the conformation of AuNPs. Both AuArg and AuCOO ligands had charged ending groups. We analyzed how the interaction of such groups with the NP surface varied moving from low to high ionic strengths in aqueous conditions. The results highlighted the capability of both AuNPs to intercalate ions and water molecules within the gold–sulfur inner shell and the tendency of ligands to bend inward, allowing the NPs to bind other species not only with the terminal ligands but also the hydrophobic alkyl chains. The bending is observed to be stronger for AuArg with respect to AuCOO, since the nanoparticle preserves quite a spherical shape, while anisotropies are evident in the case AuCOO, both at low ionic strengths and at higher ionic concentrations.

The MD simulations were then analyzed to identify the most common or recurring protein binding conformations on the AuNP of the complementary supramolecular complexes through a series of parallel simulations. The presented MD study benefited from previous BD investigations by our group [42], which produced stable and statistically relevant structures of binding complexes. This was an excellent starting point for our fully atomistic MD, showing that the binding of −30GFP to AuArg is stable up to 100 mM but more labile at the highest (200 mM) ionic strength. The protein binding changes as a function of the increased ionic strength; at low ionic strength, the binding involves the protein “engineered” barrel, while at higher ionic strength, the binding occurs with the apical parts of the protein, namely mostly the C-terminal region, and in few cases, the N-terminal region. In contrast, the instability of the binding at the highest ionic strength is not observed in the case of +36GFP interacting with AuCOO, where the binding is maintained stable and still involves a large number of charged residues of lateral beta sheets of the proteins, both at the low and the highest ionic strength.

This result provides a framework for the implementation of versatile particle–protein complexes under high salt conditions, suggesting that atomistic simulations can be a complementary tool to augment experimental studies (e.g., based on fluorescence titrations), disclosing the microscopic interactions responsible for the interfacial recognition between binding partners. The synergy between the experiments provides a solid basis for the rationalization of the intercalation of ions on the diverse surface chemistry of the AuNPs in view of future developments in the field of NP-based biosensors.

## Figures and Tables

**Table 1:** Contacting residues at distances shorter than 4 Å between AuArg and −30GFP, which were found in more than 70% of the simulation frames.

IOS	Label	Contact Residues for AuArg (>70% of Frames)
50 mM	s0	**TYR149** HIS79 HIS197 ALA225 MET76 **PHE221** GLY230 PRO73 ILE227 **ASP196** HIS75 **TYR198** GLY226 THR223 **ASP228**
	s1	ALA225 **GLU202 PHE221** ASN142 ILE227 THR223 ALA224 HIS229 **TYR198 ASP147** LEU219 **ASP228** SER200
	s2	ASN142 ILE227 ALA204 LEU219
	s3	ALA204 LEU219 **GLU202 PHE221**
100 mM	s0	**GLU202 PHE221**
	s1	ILE227 HIS229
	s2	-
	s3	HIS79 HIS75 ILE227 GLN78 HIS229
200 mM	s0	ASN210 LEU219 GLY31 LYS43 LEU42 PRO209 **GLU30** SER206 **GLU32 ASP208** THR41 VAL217
	s1	**GLU32** THR41
	s2	LEU214
	s3	-

**Table 2:** Contacting residues at distances shorter than 4 Å between AuCOO and +36GFP, which were found in more than 70% of the simulation frames.

IOS	Label	Contact Residues for AuCOO (>70% of Frames)
50 mM	s0	**LYS204** HIS146 THR41 **PHE221 LYS39** ASN168 **PHE143** ASN144 **LYS32** LEU219 **ARG202 ARG140** SER200 **ARG7** LEU218 **ARG37** SER145 ASN142 VAL217 **TYR141 LYS207** THR201 SER206 MET216 THR223 THR36 LEU205 **ARG71** ASN142 **LYS9**
	s1	**PHE221 LYS204** SER203 HIS146 ASN168 **LYS228** ASP171 **ARG202 LYS39** LEU219 **ARG140** ASN144 THR201 ASN142 GLY172 GLY226 VAL169 SER200 **ARG37** ALA225 THR223 VAL174 SER173 THR223 **LYS170 ARG71**
	s2	HIS229 SER203 HIS146 **LYS204 PHE221 LYS228 ARG202** SER145 ASN168 **LYS39** LEU219 **ARG140** LEU219 **ARG166 ARG202** ASN144 **PHE143** HIS75 GLY38 ASN142 **LYS74 LYS164 TYR141** PRO73 **LYS74 ARG37** SER206 VAL174 THR223 VAL222
	s3	**LYS204 ARG202** ILE227 LEU219 GLY31 PRO209 **LYS170 LYS39** HIS75 ASN142 SER206 GLY226 LEU218 **LYS74 ARG37 LYS30** ALA225 VAL217 **ARG37** PRO73 **LYS207** VAL217 MET216 **LYS43** LEU42 **ARG140 TYR72** PRO73 THR36 ALA224 THR223 LEU205 HIS215 THR41 **ARG71 PHE221 LYS204** ASP208 **TRP55** HIS215 **LYS32 LYS138**
100 mM	s0	**LYS124 ARG155** ASP19 **ARG107** ASP19 THR48 VAL91 SER26 GLY18 **ARG188 LYS122 PHE25 LYS24 LYS17** ASN157 ILE186 **LYS88 LYS156** THR184 PRO185 **ARG126**
	s1	VAL174 ASN196 SER203 **ARG202 LYS39 ARG166** LEU219 ASN144 THR151 SER173 GLY172 ASN142 GLY226 SER200 **TYR149** ALA225 **TYR141 LYS170 TYR149** ALA224 **ARG140** VAL169 VAL148 **PHE163 LYS162 TYR198 LYS147** THR223 LEU176 ASN168 **LYS147 PHE221 LYS204 ARG166** GLU220 **LYS228**
	s2	**LYS204 PHE221 LYS228 ARG202** ILE227 **ARG140 ARG166** HIS75 ASN142 ASN144 **PHE143** GLY226 LEU219 LYS74 ALA225 **TYR141 ARG37** THR223 LEU205 **LYS147** ASN168 HIS146 HIS229
	s3	**LYS204 LYS39** LEU219 GLY31 **ARG202 ARG7** ASN142 **ARG37** VAL217 **LYS74 ARG37** ASN144 **TYR141** THR36 LEU42 **LYS43 ARG140 ARG4 ARG71 LYS147** THR41 GLU3 **PHE221 LYS9** HIS146 **LYS77 LYS32**
200 mM	s0	THR48 **LYS124** THR47 **LYS129 LYS122 LYS105 LYS24** HIS23 **LYS103 TYR104** PRO52 GLY22 LEU135 ASN21 **ARG126 LYS50** VAL20 **LYS212** GLY49 GLY125
	s1	**ARG7** LEU219 PRO209 **LYS83** GLY31 **LYS32** ASP208 **ARG37 TYR141** LEU205 HIS215 **PHE6** LEU218 **LYS30 LYS207** VAL217 **ARG37** PRO11 THR36 **ARG71** SER206 THR41 **LYS43** THR223 **ARG71 LYS9** ALA35 **PHE221 LYS204** LEU42 THR41 **PHE221 LYS204 LYS207 LYS39**
	s2	**ARG7** LEU219 PRO209 **LYS83** GLY31 **LYS32** ASP208 **ARG37 TYR141** LEU205 HIS215 **PHE6** LEU218 **LYS30 LYS207** VAL217 **ARG37** PRO11 THR36 **ARG71** SER206 THR41 **LYS43** THR223 **ARG71 LYS9** ALA35 **PHE221 LYS204** LEU42 THR41 **PHE221 LYS204 LYS207 LYS39**
	s3	**LYS204** ASN196 HIS137 **LYS228 LYS39 ARG202** VAL174 LEU219 GLY31 PRO209 **LYS170 ARG166** SER203 ASN144 ILE227 PRO209 ASN142 **LYS164** LEU218 GLY226 VAL217 **TYR141 LYS207** ASP171 SER200 **ARG37** ASN168 THR36 ALA225 SER206 **ARG140** MET216 **LYS43** LEU42 **TYR72** PRO73 ALA224 LEU205 LEU176 LYS147 THR41 GLY172 SER173 **ARG71 TYR198** THR223 HIS229 VAL169 **PHE221** HIS229 HIS146 LEU205 HIS146 **LYS32**

## Data Availability

Not applicable.

## Supplementary Materials

Supplementary material can be found at https://www.mdpi.com/article/10.3390/ijms23042368/s1.
