# Supplementary material for: Role of Ionic Strength in the Formation of Stable Supramolecular Nanoparticle–Protein Conjugates for Biosensing
Source: Int J Mol Sci. 2022 Feb 21;23(4):2368. doi: 10.3390/ijms23042368 (PMC8874478; doi:10.3390/ijms23042368)
Supplement: Supplementary file 1 [file ijms-23-02368-s001.zip › SI_brancolini_IJMS2022.pdf]

International Journal of
Molecular Sciences

MDPI

Article

Role of Ionic Strength in the Formation of Stable Supramolecular Nanoparticle–Protein Conjugates for Biosensing

Giorgia Brancolini [1,*], Vincent M. Rotello[2], Stefano Corni[1,3]

[1] Institute of Nanoscience, CNR-NANO S3, via G. Campi 213/A, 41125, Modena, Italy; giorgia.brancolini@nano.cnr.it

[2] Department of Chemistry, University of Massachusetts, 710 North Pleasant Street, Amherst, MA 01003, USA; rotello@chem.umass.edu

[3] Department of Chemical Sciences University of Padova, via Marzolo 1, 35131 Padova, Italy; stefano.corni@unipd.it

Supporting Information

S.1 Binding Energy decomposition

In this section we present an energy decomposition analysis performed with the *rerun gromacs tool*,[1] through which we determined that -30GFP molecules tether the AuArg and the +36GFP the AuCOO, mostly through electrostatic interactions. This result provides a solid basis for the rationalization of the effect of ionic strength on the binding mechanism and stability of AuArg. The results indicate that the stability of protein-AuNP conjugates can be maintained at comparable stability even at different ionic strengths due to the number of ions trapped within the monolayer at the different ionic strength, which alter the screening due only to the solution.

Figure S1. VdW and LJ interactions between AuArg and -30GFP molecules in the 4 conformations identified at each ionic strenght.

[1] Lundborg, M., and Lindahl, E.: "Automatic GROMACS topology generation and comparisons of force fields for solvation free energy calculations", The journal of physical chemistry. B, 2015, 119, (3), pp. 810-823

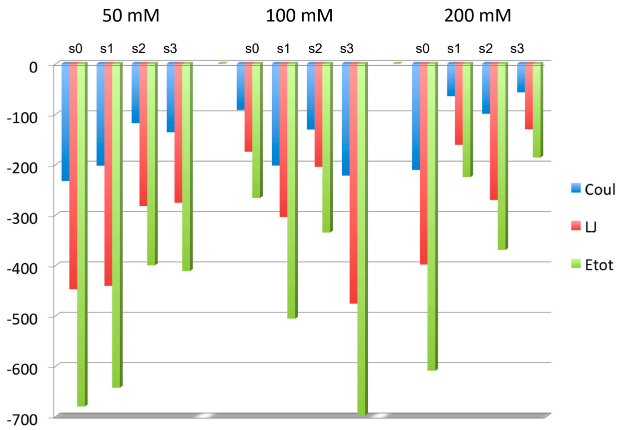

Figure S2. VdW and LJ interactions between AuCOO and +35GFP molecules in the 4 conformations identified at each ionic strenght.

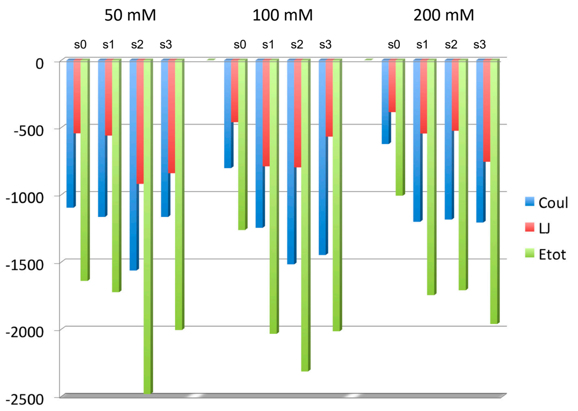

S.2. RESP charges parametrization

In this section we present the derivation of RESP charges from *ab initio* simulations.

Figure S3. RESP partial charges derivation by *ab initio* calculations for the surface atoms of the smallest interfacial repeating unit of AuArg, namely AUS-RS−AUL−SR-AUS. The SR=alkanethiol functional groups is connected to the core with a sulfur atom forming the so-called “staples” (dark and light yellow in the figure), where the sulfur atom is forming a covalent bond with one gold atom at the interface (AUL=gold ligand) and a gold atom at the surface (AUS = gold surface). Using R.E.D. server[2] the partial charges are evaluated from density functional calculations at b3lyp/6-31G*//b3lyp/6-31G* level and Connolly surface algorithm is used in MEP computation (2 stage RESP fit qwt=0.0005/0.001).

[2] Vanquelef, E., Simon, S., Marquant, G., Garcia, E., Klimerak, G., Delepine, J.C., Cieplak, P., and Dupradeau, F.Y.: ‘R.E.D. Server: a web service for deriving RESP and ESP charges and building force field libraries for new molecules and molecular fragments’, Nucleic acids research, 2011, 39, (Web Server issue), pp. W511-517

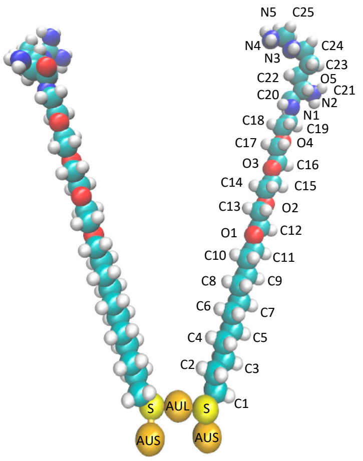

AUS	0.6200	H14	-0.0124
S	-0.5068	H14	-0.0124
AUL	0.1074	C15	0.2322
C1	-0.0036	H15	0.0138
H1	0.0172	H15	0.0138
H1	0.0172	O3	-0.5483
C2	0.7121	C16	0.3325
H2	-0.0869	H16	-0.0098
H2	-0.0869	H16	-0.0098
C3	-0.5935	C17	0.1260
H3	0.1247	H17	0.0279
H3	0.1247	H17	0.0279
C4	-0.0346	O4	-0.4676
H4	0.0030	C18	0.5630
H4	0.0030	H18	-0.0452
C5	0.3255	H18	-0.0452
H5	-0.0486	C19	-0.7527
H5	-0.0486	H19	0.2596
C6	-0.2038	H19	0.2596
H6	0.0361	N1	-0.1797
H6	0.0361	HH1	0.3160
C7	-0.0169	C20	0.6357
H7	0.0010	O5	-0.4582
H7	0.0010	C21	-0.4788
C8	0.1773	H20	0.1315
H8	-0.0299	N2	-0.4159
H8	-0.0299	HH2	0.2165
C9	-0.1561	HH2	0.2165
H9	0.0254	C22	-0.1669
H9	0.0254	H21	0.0541
C10	0.0100	H22	0.0541
H10	0.0166	C23	0.4876
H10	0.0166	H23	-0.0033
C11	0.3432	H24	-0.0033
H11	-0.0265	C24	-0.9262
H11	-0.0265	H25	0.2100
O1	-0.5743	H26	0.2100
C12	0.2887	N3	0.3205
H12	-0.0027	HH3	0.1321
H12	-0.0027	C25	-0.2253
C13	0.2998	N4	-0.0268
H13	-0.0023	HH4	0.1088
H13	-0.0023	HH4	0.1088
O2	-0.5897	N5	-0.0268
C14	0.3333	HH5	0.1088
		HH5	0.1088

Figure S4. RESP partial charges derivation by *ab initio* calculations for the surface atoms of the smallest interfacial repeating unit of AuCOO, namely AUS-RS−AUL−SR-AUS. The SR=alkanethiol functional groups is connected to the core with a sulfur atom forming the so-called "staples" (dark and light yellow in the figure), where the sulfur atom is forming a covalent bond with one gold atom at the interface (AUL=gold ligand) and a gold atom at the surface (AUS = gold surface). Using R.E.D. server[3] the partial charges are evaluated from density functional calculations at b3lyp/6-31G*//b3lyp/6-31G* level and Connolly surface algorithm is used in MEP computation (2 stage RESP fit qwt=0.0005/0.001).

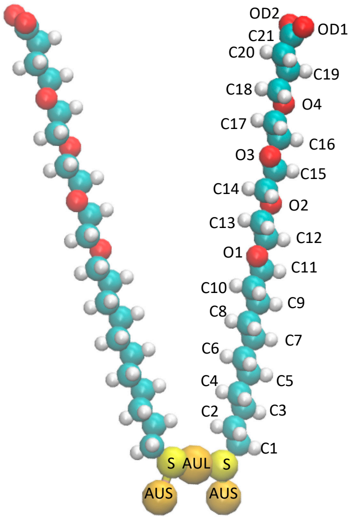

AUS	-0.3420	1H14	0.0232
S	-0.1627	2H14	0.0232
AUL	0.1278	C15	0.1680
C1	-0.0851	1H15	0.0210
1H1	0.0908	2H15	0.0210
2H1	0.0908	O3	-0.4281
C2	0.0034	C16	0.1537
1H2	-0.0157	1H16	0.0224
2H2	-0.0157	2H16	0.0224
C3	0.0976	C17	0.1938
1H3	-0.0334	1H17	0.0071
2H3	-0.0334	2H17	0.0071
C4	0.0443	O4	-0.4077
1H4	-0.0075	C18	0.1309
2H4	-0.0075	1H18	0.0042
C5	-0.0114	2H18	0.0042
1H5	-0.0072	C19	0.0182
2H5	-0.0072	1H19	0.0129
C6	0.0113	2H19	0.0129
1H6	-0.0097	C20	-0.0277
2H6	-0.0097	1H20	-0.0122
C7	0.0314	2H20	-0.0122
1H7	-0.0091	C21	0.6204
2H7	-0.0091	OD1	-0.5867
C8	0.0166	OD2	-0.5867
1H8	-0.0088		
2H8	-0.0088		
C9	0.0110		
1H9	-0.0127		
2H9	-0.0127		
C10	0.0330		
1H10	0.0097		
2H10	0.0097		
C11	0.1230		
1H11	0.0132		
2H11	0.0132		
O1	-0.4075		
C12	0.1621		
1H12	0.0204		
2H12	0.0204		
C13	0.1800		
1H13	0.0176		
2H13	0.0176		
O2	-0.4296		
C14	0.1602		

[3] Vanquelef, E., Simon, S., Marquant, G., Garcia, E., Klimerak, G., Delepine, J.C., Cieplak, P., and Dupradeau, F.Y.: 'R.E.D. Server: a web service for deriving RESP and ESP charges and building force field libraries for new molecules and molecular fragments', Nucleic acids research, 2011, 39, (Web Server issue), pp. W511-517
